# Dental Education

**Published:** 1872-01

**Authors:** 


### Editorial.

### DENTAL EDUCATION.

Any one proposing to enter the dental profession, and take upon himself its duties, responsibilities, and labors, should possess, first of all, a natural, inherent fitness and adaptation for the work. Not only is this applicable to the practical duties involved, but to the preparation for the work. To constitute a good dentist, both a good student and a good manipulator, is important. Many in the profession are good students, but very defective manipulators, dependent, oftentimes, upon a want of proper training, but more frequently upon deficient natural endowment. By great industry, perseverance, and special advantages, this may in some instances be largely overcome; in others, the deficiency is irremediable.

Industrious students are often averse to manual labors; and, on the other hand, those who incline to physical efforts are but little disposed to purely mental effort or labor.

Both these classes are found in our profession, and in very considerable numbers, too; and, as a whole, their success is not what it should be for the best interests of all concerned. Presuming that there is a good degree of natural endowment, let us inquire: What should be the aim in an educational aspect? What is education? It is not a mere storing up in the mind an accumulation of facts, principles or knowledges: These are important, it is true, but they are only the instruments that are to be subsequently used; but it is also a *drawing out, developing, strengthening and training* the mental faculties, so that the materials that may be treasured up may be employed most efficiently. The things employed in this training are not necessarily all to be retained in special remembrance. Having served their purpose, they should be laid aside as no longer needed.

The dental student should possess, as preparatory to his professional studies, a thorough literary, classic and scientific education. Then, and not till then, is he prepared to undertake and pursue his professional studies with profit and the greatest good; only by that means can he be intellectually prepared to grapple with and overcome the subjects that will be presented for his consideration. As in almost all other departments of human occupation, superficiality has been the bane of our profession; and that, too, with the approval, or at least the silent consent, of those who ought to have raised a warning, if not a condemning voice. We have too long silently acquiesced in the general impression that a little manipulative skill is all that is necessary to constitute a dentist.

After a proper elementary education, every department of medical science should receive the dental student's thorough investigation. Especially should *anatomy*, *physiology*, *histology*, *pathology*, *chemistry and therapeutics* be studied up to the point of their present development. We do not mean by this that he should cumber the memory with all difficult names and technicalities, but that the knowledge of these branches should enter so fully into his apprehension that mere names would dwindle into utter insignificance. This knowledge should so enter in and make up the professional personality, that its removal or loss would be impossible. In the thorough assimilation and appropriation of this professional food will the well developed physician and dentist be produced, with a strength and power sufficient for every emergency.

The professional attainments of every student will in every instance present the impress of him who in the beginning gives it direction. Every kind begets its like. Ignorance tends to perpetuate itself; and too often it succeeds in blinding others as to its true character.

---

As WILL be seen by reference to the proceedings of the Ohio State Dental Society, on page 39 of the present number of the REGISTER, at the last meeting of the State Society, a committee of five was appointed, whose duty it shall be to take under advisement, and pursue such course as shall seem to be demanded by the exigencies of the approaching rubber contest. We understand that consultation has been had by the committee, but no definite line of action yet decided upon. All communications upon the subject should be addressed to some member of the committee, as they will have more information on the subject than any one else.

